# Occupational stress, coping strategies, and associated factors among primary healthcare workers in Vietnam

**DOI:** 10.1371/journal.pone.0349770

**Published:** 2026-05-20

**Authors:** Han Thi Ngoc Nguyen, Phi Hoang Nguyen, Han Thi Ngoc Nong, Ly Trieu Vo, Dung Dang Nguyen, Nhi Thi Bui, Yen Nhi Thi Nguyen, Giao Huynh

**Affiliations:** 1 Infection Control Department, University Medical Center Ho Chi Minh City, Ho Chi Minh City, Vietnam; 2 Faculty of Public Health, University of Medicine and Pharmacy at Ho Chi Minh City, Ho Chi Minh City, Vietnam; 3 Department of Infectious Diseases E, Hospital for Tropical Diseases, Ho Chi Minh City, Vietnam; 4 Administrative Department, Tan Phu Hospital, Ho Chi Minh City, Vietnam; 5 Department of Obstetrics & Gynecology, Stanford University School of Medicine, Stanford, California, United States of America; Alexandria University Faculty of Nursing, EGYPT

## Abstract

**Background:**

Occupational stress is a growing concern among healthcare workers (HCWs), particularly in primary care settings where responsibilities are multifaceted and resources are limited.

**Objective:**

This study aimed to determine the proportion of perceived stress levels, identify the coping strategies employed, and explore sociodemographic, familial, and occupational factors associated with stress among HCWs in a district-level health system in Ho Chi Minh City, Vietnam.

**Methods:**

A cross-sectional study was conducted from 19 February 2025–31 March 2025 involving 212 HCWs selected through total population sampling. Data were collected using a structured self-administered questionnaire comprising the 10-item Perceived Stress Scale (PSS-10) and the Brief COPE inventory. Descriptive statistics summarized participant characteristics and stress levels. Comparisons were performed using independent t-tests and one-way ANOVA, while Spearman’s rank correlation explored associations between continuous variables. Variables with p < 0.20 in univariate analysis were included in a multivariate linear regression model with backward elimination to identify independent predictors of stress. Multicollinearity was assessed using the Variance Inflation Factor (VIF).

**Results:**

The mean PSS-10 score was 16.7 ± 2.3, with 93.4% of participants experiencing moderate stress and 6.6% low stress. The most frequently endorsed coping strategies were denial (5.8 ± 1.2), behavioral disengagement (5.4 ± 1.4), and substance use (5.0 ± 1.1). Multivariate analysis revealed that younger age, having chronic diseases, and being a physician were independently associated with higher perceived stress.

**Conclusion:**

The study reveals a high prevalence of moderate stress among HCWs in primary care, with frequent use of ineffective coping strategies. Interventions should be tailored to promote effective coping, reduce occupational burdens, and provide targeted support to vulnerable subgroups.

## Introduction

Healthcare professionals face high workloads and resource shortages in emotionally demanding environments on an everyday basis [1]. This can lead to stress, which is defined as a psychological state of worry or mental tension with potentially harmful effects on physical and emotional well-being, according to the World Health Organization [2]. By extension, work-related stress is called occupational stress [3,4] . In Vietnam, the prevalence of occupational stress among HCWs ranges from 26.7% to 40.6%, primarily at mild to moderate levels [5–7]. This statistic sharply increased during the COVID-19 pandemic, with roughly 34% experiencing moderate and approximately 30% severe stress [8]. Previous studies show that prolonged stress among HCWs is associated with burnout, increased risks of errors, and diminished quality of patient care. Not addressing prolonged stress may lead to or worsen anxiety, depression, sleep disturbances, and emotional dysregulation [8–10]. The problem of stress has been explored in various populations, including athletes [11], students [12], and patients [13]. Among HCWs, risk factors for stress include age, education level, marital status, work conditions, and comorbid mental health conditions [14–17]. In Vietnam, studies addressing HCWs’ occupational stress were mostly conducted in hospitals [5–7,14,15], while little is known about the stress levels of HCWs working at the primary care level. Therefore, this study aims to address the gap in understanding stress among this particular population in Ho Chi Minh City, Vietnam, to inform population- and setting-specific interventions.

Several explanatory theories have been developed to evaluate perceived stress. The Richard Lazarus and the Susan Folkman Transactional Model of Stress emphasize that cognitive appraisal shapes subjective stress experiences [18]. The Robert Karasek Job Demand-Control model focuses on the impacts of high job demands and low autonomy [19]. The Johannes Siegrist Effort-Reward Imbalance model addresses the mismatches between effort and rewards [20]. The Stevan Hobfoll Conservation of Resources theory emphasizes resource loss and protection as central to stress responses [21]. Chronic work stress theory discusses the health impact of prolonged exposure to stressors [22]. Lastly, the Transactional Model provides a unifying framework for understanding how individuals perceive and respond to stressors. Therefore, this study uses that model as its theoretical framework.

The Perceived Stress Scale (PSS) is recognized as the gold standard for assessing perceived stress and evaluating the degree to which individuals perceive their life situations as unpredictable, uncontrollable, or overwhelming [23–26]. Unlike objective checklists of life events [27,28], the PSS captures the subjective appraisal of stress specifically and whether they perceive external demands as exceeding their adaptive capacity [23]. The PSS-10 is a brief, psychometrically robust, and widely used tool that comprises perceived helplessness and perceived self-efficacy [29,30]. Moreover, the Brief Coping Orientation to Problem Experienced (Brief COPE) scale is also widely used to assess coping strategies. It has been validated across a range of populations and contexts with an acceptable internal consistency across subscales (median Cronbach’s α = 0.75, ranging from 0.54 to 0.91) [26,31,32]. Coping strategies in the Brief COPE are categorized as problem-focused, emotion-focused, or avoidant. Research has shown that adaptive coping mechanisms are associated with lower stress levels and, therefore, improved psychological resilience [33,34]. These mechanisms include seeking social support, problem-solving, and positive reframing. In contrast, avoidance, denial, or substance use are linked to increased stress and burnout risks [34].

## Materials and methods

### Study design and population

This cross-sectional study targeting healthcare workers at 12 health stations serving approximately 485,000 residents in Ho Chi Minh City, Vietnam, where staff are responsible for a wide range of public health duties- including disease prevention, health promotion, and outpatient care- despite substantial workloads and limited staffing [35].

The sample size was calculated using the formula for estimating a population proportion, based on the following assumptions: a prevalence of stress among HCWs (p) of 15.4% as reported in prior research of Bui HC et al [36] a Z-score ($Z_{1-\frac{\alpha }{2}}$) of 1.96 corresponding to a Type I error (α) of 0.05 for a 95% confidence level, and an estimation error (d) of 5% [37].


$$\begin{array}{c} n=\overset{2}{\underset{1-\frac{\alpha }{2}}{Z}}\frac{(1-p)p}{d^{2}} \end{array}$$

Therefore, the minimum required sample size for the study was 201 participants to ensure adequate statistical power.

The study population included all HCWs from the 12 health stations in Ho Chi Minh City, Vietnam. The HCWs including physicians, pharmacists, physical assistants, nurses, midwives, technicians, administrative staff and support staff.

A convenience sampling strategy was considered on all eligible HCWs were invited to participate between 19 February 2025 and 31 March 2025. Eligible participants were healthcare workers who had at least six months of continuous service and provided informed consent to participate in the study. All participants were informed about the objective of the study and completed the consent form before participating. Those absent on two or more occasions during data collection or who failed to complete the entire survey questionnaire were excluded. Finally, a total of 212 HCWs were recruited using total population sampling.

### Data collection

A self-administered questionnaire composed of three main sections: sociodemographic characteristics, the 10-item version of the PSS, and the Brief COPE instrument. The first section included (1) **sociodemographic** included age, gender, ethnicity (categorized as Kinh, which is the majority ethnic group in Vietnam, accounting for approximately 85.3% of the population [38], or other), religion, educational level, family-related variables which comprised household composition, relationship quality with cohabitants, alcohol consumption, smoking; (2) **clinical variables** included the presence of chronic conditions such as hypertension, diabetes, asthma, chronic obstructive pulmonary disease (COPD), previously diagnosed psychological disorders; (3) **occupational characteristics** such as job title, years of experience, perceived task overload, and perceived suitability of the working environment. The second section included the PSS-10 scale to assess perceived stress experienced over the past month [39]. The scale comprises ten items, including six negatively worded statements (items 1, 2, 3, 6, 9, and 10) that measure perceived helplessness or feeling unable to act effectively, and four positively worded items (items 4, 5, 7, and 8) that assess perceived self-efficacy, or the ability to cope effectively with stressors. Responses are rated on a 5-point Likert scale ranging from 0 (“Never”) to 4 (“Very often”), with the four positively worded items reverse-scored to ensure that higher total scores reflect greater perceived stress. The total score ranges from 0 to 40. As the PSS-10 does not have standardized cut-off values were specified by the scale’s developers, stress levels were categorized into low (0−13), moderate (14−26), and high (27−40) based on thresholds commonly used in previous studies [40–42]. The PPS-10 has been using widely in different populations, including Vietnam. Dao-Tran, TH., et al translated and assessed the psychometric properties of instrument, demonstrating its acceptable validity and reliablity [30].

The third section is the Brief COPE scale scores each subscale separately to capture the diversity of coping mechanisms [31]. This 28-item self-report instrument evaluates 14 distinct coping domains, each represented by two items. These domains include both adaptive strategies, including active coping, planning, positive reframing, acceptance, seeking emotional or instrumental support, humor and religion, and less adaptive strategies, including self-distraction denial, venting, self-blame, behavioral disengagement, and substance use. Participants rated the frequency of using each strategy on a 4-point Likert scale ranging from 1 (“I haven’t been doing this at all”) to 4 (“I’ve been doing this a lot”). The Brief COPE has been translated and evaluated for reliability and validity in Vietnam, demonstrating high internal consistency with Cronbach’s α = 0.85 and good construct validity [43].

### Data analysis

Completed questionnaires were reviewed and exported to STATA version 17.0 for analysis. The normality of continuous variables was assessed using the Shapiro-Wilk test. Categorical variables were summarized as frequencies and percentages, while continuous variables were reported as means ± standard deviations (SD) or medians with interquartile ranges (IQR), depending on distribution. The homogeneity of variances was evaluated using Levene’s test. Independent t-tests and one-way ANOVA were used for normally distributed variables with homogeneous variances. For non-normally distributed data, non-parametric tests (Mann-Whitney U test or Kruskal-Wallis test) were used. Spearman’s correlation was used to assess asscociations betweennon-normally distributed or non-linear continuous variables.

Linear regression analysis was conducted to assess associations between independent variables and PSS-10 scores. Model assumptions, including linearity, normality of residuals, homoscedasticity, and independence of errors (assessed using the Durbin-Watson statistic), were evaluated. Variables with p < 0.20 in univariate analysis were included in the multivariable model, and backward elimination was applied to identify independent predictors. Multicollinearity was assessed using the Variance Inflation Factor (VIF), with values >10 considered problematic; no variable exceeded this threshold. Statistical significance was set at p < 0.05. Results from the final model were reported as adjusted coefficients (β) with corresponding 95% confidence intervals (CI).

### Ethical considerations

Ethical approval was obtained from the Institutional Review Board of the University of Medicine and Pharmacy at Ho Chi Minh City (853/ĐHYD-HĐĐĐ, 18 February 2025). This study was conducted in accordance with the Declaration of Helsinki. All participants provided written informed consent prior to data collection. Confidentiality and anonymity were strictly maintained throughout the study. Participants were informed of their right to withdraw at any time without consequences.

## Results

A total of 212 questionnaire responses were obtained, and the sociodemographic is presented in Table 1. Most participants were male (61.3%), of Kinh ethnicity (93.4%), and affiliated with a religion (83.0%). A bachelor’s degree was held by 67.9% of them, while 37.3% reported alcohol consumption and 13.7% reported smoking. Most lived with others (90.1%) and perceived their relationships with relatives as very good (57.5%) or good (29.7%). The most common professional roles were Physician assistant/Nurse/Midwife/Technician/administrative staff/support staff (54.7%). Participants had a median of 10 years of experience (IQR: 3–18) and nearly three-quarters rated their work environment as somewhat unsuitable.

**Table 1:** Characteristics of the participants (N = 212).

Characteristics	Values	Frequency (Percentage)
**Sociodemographic features**		
Age (year) ^*^	37.5 (31–46)	
Gender	Male	82 (61.3)
	Female	130 (38.7)
Ethnicity	The Kinh people	198 (93.4)
	Others	14 (6.6)
Religion	Yes	176 (83.0)
Level of education	Vocational/College level	47 (22.2)
	Bachelor’s degree	144 (67.9)
	Postgraduate degree	21 (9.9)
Alcohol consumption	Yes	79 (37.3)
Smoking	Yes	29 (13.7)
Living with others	Yes	191 (90.1)
Household members	Spouse	120 (56.6)
	Children	125 (59.0)
	Biological parents or Parents-in-law	68 (32.1)
	Other relatives	41 (19.3)
Quality of relationships with relatives	Very good	122 (57.5)
	Good	63 (29.7)
	Not good	27 (12.8)
**Clinical features**		
Chronic disease	Yes	27 (12.7)
Psychological disorders	Yes	7 (3.3)
**Occupational features**		
Job title	Physician	65 (30.7)
	Pharmacist	31 (14.6)
	**Others**	**116 (54.7)**
	Physician assistant	21(9.9)
	Nurse	25(11.8)
	Midwife	22(10.4)
	Techinician	21(9.9)
	Administrative staff	15(7.1)
	Support staff	12(5.7)
Years of experience (year) ^*^	10 (3–18)	
Heavy workload	Never	51 (24.0)
	Sometimes	131 (61.8)
	Often	22 (10.4)
	Always	8 (3.8)
Work environment	Very suitable	40 (18.9)
	Suitable	5 (2.3)
	Somewhat unsuitable	156 (73.6)
	Completely unsuitable	11 (5.2)

* Median (IQR).

Table 2 depicts the items’ score of the perceived stress of participants. As shown in Table 3, the overall mean PSS-10 score was 16.7 ± 2.3, indicating a predominance of moderate stress levels with 93.4% experiencing moderate stress.

**Table 2:** Descriptive statistics of perceived stress (PSS-10) by items (N = 212).

Subscales	Items	Mean ± SD
In the last month, how often have you…		
Perceived helplessness	been upset because of something that happened unexpectedly?	1.4 ± 0.8
	felt that you were unable to control the important things in your life?	1.7 ± 1.1
	felt nervous and stressed?	1.8 ± 0.9
	found that you could not cope with all the things that you had to do?	2.1 ± 1.0
	been angered because of things that happened that were outside of your control?	1.5 ± 0.7
	felt difficulties were piling up so high that you could not overcome them?	1.5 ± 1.0
Lack of self-efficacy	felt confident about your ability to handle your personal problems?	2.1 ± 0.8
	felt that things were going your way?	2.2 ± 0.6
	been able to control irritations in your life?	1.1 ± 0.8
	felt that you were on top of things?	1.5 ± 0.9

**Table 3:** Descriptive statistics of perceived stress (PSS-10) by levels (N = 212).

Stress levels	Frequency	Percentage	PSS-10 score Mean ± SD
Low stress	14	6.6	16.7 ± 2.3
Moderate stress	198	93.4	
High stress	0	0	

Coping strategies employed by participants are summarized in Table 4. Among the 14 subscales of the Brief COPE inventory, the most frequently used strategies were denial (5.8 ± 1.2), behavioral disengagement (5.4 ± 1.4), and active coping (5.3 ± 1.4), whereas the lowest mean scores were observed for planning (3.5 ± 1.3) and venting (3.8 ± 1.4).

**Table 4:** Descriptive statistics of coping strategies (Brief COPE) (N = 212).

Strategies of Brief COPE	Items	Mean ± SD
Active coping	I’ve been concentrating my efforts on doing something about the situation I’m in. I’ve been taking action to try to make the situation better.	5.3 ± 1.4
Instrumental support	I’ve been getting help and advice from other people. I’ve been trying to get advice or help from other people about what to do.	4.5 ± 1.3
Positive reframing	I’ve been trying to see it in a different light, to make it seem more positive. I’ve been looking for something good in what is happening.	4.4 ± 1.1
Planning	I’ve been trying to come up with a strategy about what to do. I’ve been thinking hard about what steps to take.	3.5 ± 1.3
Emotional support	I’ve been getting emotional support from others. I’ve been getting comfort and understanding from someone.	4.6 ± 1.3
Acceptance	I’ve been accepting the reality of the fact that it has happened. I’ve been learning to live with it.	5.0 ± 1.4
Humor	I’ve been making jokes about it. I’ve been making fun of the situation.	4.7 ± 1.3
Religion	I’ve been trying to find comfort in my religion or spiritual beliefs I’ve been praying or meditating	4.7 ± 1.2
Self-blame	I’ve been criticizing myself. I’ve been blaming myself for things that happened	4.6 ± 1.2
Self-distraction	I’ve been turning to work or other activities to take my mind off things. I’ve been doing something to think about it less, such as going to movies, watching TV, reading, daydreaming, sleeping, or shopping.	4.4 ± 1.3
Denial	I’ve been saying to myself “this isn’t real”. I’ve been refusing to believe that it has happened.	5.8 ± 1.2
Venting	I’ve been saying things to let my unpleasant feelings escape. I’ve been expressing my negative feelings.	3.8 ± 1.4
Substance use	I’ve been using alcohol or other drugs to make myself feel better I’ve been using alcohol or other drugs to help me get through it.	5.0 ± 1.1
Behavioral disengagement	I’ve been giving up trying to deal with it. I’ve been giving up the attempt to cope.	5.4 ± 1.4

Bivariate analysis in Table 5 revealed a significant negative correlation between age and stress levels (r = −0.150, p = 0.029), while self-blame was positively correlated with stress (r = 0.148, p = 0.031). Instrumental and emotional support showed marginal associations with stress but did not reach statistical significance.

**Table 5:** Factors related to occupational stress (N = 212).

Independent variable	Perceived stress scale	t/F	p-value
	Mean ± SD or Coefficient		
**Sociodemographic features**			
Age (year)	−0.150		**0.029** ^*^
Gender			
Male	16.7 ± 2.2	−0.107	0.915^†^
Female	16.7 ± 2.4		
Ethnicity			
The Kinh people	16.6 ± 2.3	1.102	0.272^†^
Others	17.3 ± 2.5		
Religion			
Yes	16.6 ± 2.3	1.095	0.275^†^
No	17.1 ± 2.2		
Level of education			
Vocational/College level	16.5 ± 2.6	0.30	0.744^‡^
Bachelor’s degree	16.8 ± 2.2		
Postgraduate degree	16.8 ± 2.1		
Alcohol consumption			
Yes	16.9 ± 2.2	−1.095	0.275^†^
No	16.6 ± 2.3		
Smoking			
Yes	16.9 ± 2.1	−0.581	0.561^†^
No	16.7 ± 2.3		
Living with others			
Yes	16.7 ± 2.2	−0.164	0.869^†^
No	16.6 ± 2.9		
Living with spouse			
Yes	16.5 ± 2.1	1.626	0.106^†^
No	17.0 ± 2.6		
Living with children			
Yes	16.5 ± 2.3	1.527	0.128^†^
No	16.9 ± 2.3		
Living with biological parents or parents-in-law			
Yes	16.8 ± 2.3	−0.414	0.679^†^
No	16.6 ± 2.3		
Living with other relatives			
Yes	17.1 ± 2.1	−1.382	0.168^†^
No	16.6 ± 2.4		
Quality of relationships with relatives			
Very good	16.8 ± 2.7	1.00	0.368^‡^
Good	17.0 ± 2.2		
Not good	16.5 ± 2.3		
**Clinical features**			
Chronic disease			
Yes	17.4 ± 2.0	−1.711	0.089^†^
No	16.6 ± 2.3		
Psychological disorders			
Yes	17.4 ± 2.1	−0.848	0.398^†^
No	16.7 ± 2.3		
**Occupational features**			
Job title			
Physician	17.3 ± 2.1	2.82	0.062^‡^
Pharmacist	16.5 ± 2.2		
Others	16.4 ± 2.4		
Years of experience (year)	−0.088		0.201^*^
Heavy workload			
Never	16.7 ± 2.1	0.11	0.956^‡^
Sometimes	16.6 ± 2.4		
Often	16.8 ± 1.8		
Always	17.1 ± 3.3		
Work environment			
Very suitable	16.7 ± 2.7	1.28	0.282^‡^
Suitable	18.0 ± 2.3		
Somewhat unsuitable	16.6 ± 2.2		
Completely unsuitable	17.6 ± 2.3		
**Strategies of Brief COPE**			
Active coping	0.099		0.149^*^
Instrumental support	0.125		0.068^*^
Positive reframing	0,081		0.243^*^
Planning	0.083		0.227^*^
Emotional support	0.113		0,102^*^
Acceptance	0.063		0.363^*^
Humor	0.048		0.488^*^
Religion	0.066		0.335^*^
Self-blame	0.148		**0.031** ^*^
Self-distraction	0.034		0.618^*^
Denial	0.082		0.236^*^
Venting	0.054		0.431^*^
Substance use	0.033		0.632^*^
Behavioral disengagement	−0.006		0.927^*^

* Spearman correlation coefficient; † T-test; ‡ Oneway ANOVA.

Table 6 presented the results of the correlational analysis. Multivariate linear regression showed that age remained a significant negative predictor of stress (β = −0.03; 95% CI: −0.06 to −0.01; p = 0.043). Participants with chronic diseases exhibited significantly higher stress levels (β = 1.19; 95% CI: 0.23–2.15; p = 0.016). Moreover, physicians reported significantly greater stress compared to individuals in other occupational groups (β = 0.72; 95% CI: 0.01–1.43; p = 0.046).

**Table 6:** Multiple Linear Regression Analysis of stress and participant characteristics (N = 212).

Independent variables	Univariate analysis		Multivariate analysis	
	β(95% CI)	p-value	Adjusted β (95% CI)	p-value
Age (year)	−0.03 (−0.06 to 0.00)	0.058	−0.03 (−0.06 to −0.01)	**0.043**
Chronic disease (yes)	0.81 (−0.12 to 1.75)	0.089	1.19 (0.23 to 2.15)	**0.016**
Living with a spouse (yes)	−0.53 (−1.16 to 0.09)	0.097	–	–
Living with children (yes)	−0.49 (−1.12 to 0.14)	0.128	–	–
Living with other relatives (yes)	0.55 (−0,24 to 1.35)	0.168	–	–
Job title				
Physician Pharmacist Others	0.82 (0.12 to 1.52) 0.04 (−0.87 to 0.96) *ref*	**0.022** 0.924	0.72 (0.01 to 1.43) 0.18 (−0.73 to 1.08) *ref*	**0.046** 0.701
Active coping	0.14 (−0.07 to 0.36)	0.193	–	–
Instrumental support	0.21 (−0.04 to 0.46)	0.104	–	–
Emotional support	0.21 (−0.03 to 0.45)	0.081	–	–
Self-blame	0.26 (−0.00 to 0.52)	0.051	–	–

ref: Reference group; Bold values indicate statistically significant differences (p < 0.05) in the univariate and multivariable linear regression models; A “-” symbol in the blank cells to show that these variables were analyzed but were not significant in the final multivariate model.

## Discussion

This study demonstrated that the majority of healthcare workers experienced moderate levels of perceived stress. These results differ from previous studies that reported the prevalence of stress at relatively low levels, ranging from 15.4% to 21.7% [15,36]. During the COVID-19 pandemic, Yubonpunt P et al and Michalak M et al reported stress levels were higher, at 41.97% and 47%, respectively [34,44]. The differences across these studies can be explained by the diversity in instruments, cut-off criteria, pandemic exposure, work environment, and cultural attitudes toward stress expression. Similarly, studies using the EASE scale and Occupational Stress Index have reported substantial levels of stress among HCWs [33,45]. Although primarily measurement-based, these findings can be interpreted within broader frameworks beyond the Lazarus and Folkman transactional model of stress, particularly the Conservation of Resources theory and the Job Demand-Control model. With 93.4% of HCWs reporting moderate stress, it is likely that there is an issue within the healthcare environment. This is consistent with the Job Demand-Control model and the Effort-Reward Imbalance model, which emphasize the effects of high job demands, low control, and insufficient rewards [19,20]. From the perspective of the Conservation of Resources theory, this may also reflect cumulative resource depletion over time [21]. Such widespread stress has important implications for workforce well-being, quality of care, and patient safety that necessitate the need for system-level interventions.

Coping strategies mediate between stressors and responses and are categorized as adaptive coping (active problem-solving, acceptance, positive reframing) or maladaptive coping (avoidance, withdrawal, substance use). We observed a strong association between perceived stress level and the type of coping strategies used. Interestingly, HCWs with higher stress levels relied more heavily on avoidant strategies such as denial, behavioral disengagement, and substance use, while still concurrently engaging in adaptive approaches. This concurrent use of coping styles suggests stress dynamism; however, the overall psychological outcome is more likely shaped by the predominance one coping strategy category over the other. This finding is consistent with the study by Aryal S et al, conducted among community health workers in Mangalore Taluk, Karnataka, which reported that avoidant coping strategies, particularly self-distraction, denial, substance use, behavioral disengagement, and self-blame, were significantly associated with occupational stress [33]. Similarly, Gonzalez Delgado M et al found that those who relied on avoidance and disengagement coping had significantly higher rates of acute stress and depression, greater declines in mental well-being, and the highest levels of intention to leave the profession [45]. This reiterates that while avoidant coping may provide short-term relief, its overuse is closely linked to worsened psychological health if not balanced by adaptive coping.

On the other hand, the previous research by Folkman and Lazarus has emphasized that problem-focused coping strategies can enhance perceived control, reduce anxiety, and promote psychological recovery more effectively than strategies focused on emotional relief [46]. However, a noteworthy observation in our study is that some individuals use active coping, planning, and seeking emotional or instrumental support but remain at high levels of perceived stress. Using adaptive strategies may not guarantee stress reduction if those strategies are not implemented effectively; for example, a healthcare worker may attempt to seek help but encounter negative reactions from colleagues or supervisors. In such cases, a potentially helpful coping strategy may backfire and cause increased frustration and emotional burden.

We found that older healthcare workers had significantly lower levels of perceived stress. This finding is consistent with that of Tran et al and Aldarmasi MA et al., who reported that HCWs under 30 years experience higher levels of stress compared to their older colleagues [15,47]. This may be because of greater psychological resilience among older individuals. It is therefore possible that age is as a protective factor against occupational stress. Previous studies conducted in Germany [48] and Ethiopia [49] further illustrate that newly graduated doctors and nurses are more vulnerable to stress due to the onset of demanding work schedules and limited clinical experience [50]. In contrast, older staff may possess more refined coping mechanisms, better emotional regulation, and greater familiarity of workplace dynamics [51]. Therefore, interventions aimed at reducing stress should prioritize younger HCWs’ needs.

Consistent with findings from Nguyen et al and previous studies conducted in Saudi Arabia, we also identified a statistically significant association between the presence of chronic comorbidities and elevated occupational stress levels [14,16]. One possible explanation is that individuals with chronic health conditions may be more sensitive to health-related stressors, such as fear of disease progression, fatigue, or concerns about access to care. These can in turn amplify their overall stress perception. At the same time, these individuals might not necessarily report higher stress related to work environment or interpersonal relationships, potentially resulting in variability across stress domains.

In our multivariate model, physicians had significantly higher perceived stress scores compared to other healthcare roles, which contrasts with the study by Tran et al, where nurses and administrative staff reported higher stress levels [15]. This discrepancy may be explained by differences in workplace context. Our study was conducted in health stations, where physicians are responsible for public health, administrative tasks, and other duties beyond clinical care. In contrast, the study by Tran et al took place in a large hospital with more structured role divisions and institutional resources. These findings highlight that interventions to reduce occupational stress should consider organizational structure, role expectations, and system-level support.

This study has several strengths. First, it offers a comprehensive reflection of perceived stress and coping strategies within the context of urban primary care by surveying HCWs across 12 different health stations. Second, by focusing on primary healthcare staff, a group frequently faced with high pressure, this study provides practical insights with high relevance for workforce support and policy planning. Additionally, the inclusion of multiple domains such as individual, family, and occupational factors in the analysis allows for a more holistic understanding of stress and its determinants, thereby better informing interventions. However, some limitations can be considered in this study. First, the use of a self-administered questionnaire may introduce recall bias or social desirability bias, potentially affecting the accuracy of self-reported stress and coping behaviors. Second, a convenience sampling may reduce the representativeness of the sample and limit the generalizability of the findings. Third, the study did not assess comorbid mental health conditions such as depression or anxiety, which limits its ability to evaluate the broader picture of psychological well-being.

## Conclusion

This study highlights a substantial burden of perceived stress among healthcare workers at the primary care level in a densely populated district of Ho Chi Minh City, Vietnam, with the vast majority experiencing moderate stress levels. Ineffective coping strategies, particularly denial, behavioral disengagement, and substance use, were prominently reported and associated with higher stress, while the protective effects of adaptive coping appeared to be influenced by situational and organizational factors. The reveal of age, chronic illness, and job title as significant predictors of stress emphasize that the interaction between personal, health-related, and occupational determinants are complex. These findings call for targeted mental health interventions that not only promote adaptive coping mechanisms but also address structural contributors to stress, such as workload distribution and systemic support.

## Supporting information

S1 Dataset PSS data. (XLS)

S2 Checklist STROBE checklist. (DOCX)
