# Supplementary material for: Occupational stress, coping strategies, and associated factors among primary healthcare workers in Vietnam
Source: PLoS One. 2026 May 20;21(5):e0349770. doi: 10.1371/journal.pone.0349770 (PMC13189326; doi:10.1371/journal.pone.0349770)
Supplement: S2 Checklist — (DOCX) [file pone.0349770.s002.docx]

**STROBE Statement—checklist of items that should be included in reports of observational studies**

|  | Item No. | Recommendation | No. |
| --- | --- | --- | --- |
| **Title and abstract** | 1 | (*a*) Indicate the study’s design with a commonly used term in the title or the abstract | Line 15-43 |
|  |  | (*b*) Provide in the abstract an informative and balanced summary of what was done and what was found | Line 15-43 |
| Introduction | | | |
| Background/rationale | 2 | Explain the scientific background and rationale for the investigation being reported | Line 44-61 |
| Objectives | 3 | State specific objectives, including any prespecified hypotheses | Line 59-61 |
| Methods | | | |
| Study design | 4 | Present key elements of study design early in the paper | Line 87-108 |
| Setting | 5 | Describe the setting, locations, and relevant dates, including periods of recruitment, exposure, follow-up, and data collection | Line 87-91  Line 99-108 |
| Participants | 6 | *Cross-sectional study*—Give the eligibility criteria, and the sources and methods of selection of participants | Line 103-108 |
| Variables | 7 | Clearly define all outcomes, exposures, predictors, potential confounders, and effect modifiers. Give diagnostic criteria, if applicable | Line 110- 141 |
| Data sources/ measurement | 8* | For each variable of interest, give sources of data and details of methods of assessment (measurement). Describe comparability of assessment methods if there is more than one group | Line 110-141 |
| Bias | 9 | Describe any efforts to address potential sources of bias | Line 105-108 |
| Study size | 10 | Explain how the study size was arrived at | Line 88 - 91 |

| Quantitative variables | 11 | Explain how quantitative variables were handled in the analyses. If applicable, describe which groupings were chosen and why | Line 92-98 |
| --- | --- | --- | --- |
| Statistical methods | 12 | (*a*) Describe all statistical methods, including those used to control for confounding | Line 143-160 |
|  |  | (*b*) Describe any methods used to examine subgroups and interactions | Line 143-160 |
|  |  | (*c*) Explain how missing data were addressed | Line 143-160 |
|  |  | (*d*) *Cross-sectional study*—If applicable, describe analytical methods taking account of sampling strategy | Line 143-160 |
|  |  | (*e*) Describe any sensitivity analyses | Line 143-160 |
| Participants | 13* | (a) Report numbers of individuals at each stage of study—eg numbers potentially eligible, examined for eligibility, confirmed eligible, included in the study, completing follow-up, and analysed | Line 169 |
|  |  | (b) Give reasons for non-participation at each stage | Line 169 |
|  |  | (c) Consider use of a flow diagram | We didn’t use flow diagram in this manuscript |
| Descriptive data | 14* | (a) Give characteristics of study participants (eg demographic, clinical, social) and information on exposures and potential confounders | Line 177 |
|  |  | (b) Indicate number of participants with missing data for each variable of interest | Line 177 |
| Outcome data | 15* | *Cross-sectional study—*Report numbers of outcome events or summary measures | Line 183 |
| Main results | 16 | (*a*) Give unadjusted estimates and, if applicable, confounder-adjusted estimates and their precision (eg, 95% confidence interval). Make clear which confounders were adjusted for and why they were included | Line 201 |
|  |  | (*b*) Report category boundaries when continuous variables were categorized | Line 168- 204 |
|  |  | (*c*) If relevant, consider translating estimates of relative risk into absolute risk for a meaningful time period | Line 201 |

| Other analyses | 17 | Report other analyses done—eg analyses of subgroups and interactions, and sensitivity analyses | Line 201 |
| --- | --- | --- | --- |
| Discussion | | | |
| Key results | 18 | Summarise key results with reference to study objectives | Line 205- 273 |
| Limitations | 19 | Discuss limitations of the study, taking into account sources of potential bias or imprecision. Discuss both direction and magnitude of any potential bias | Line 274-286 |
| Interpretation | 20 | Give a cautious overall interpretation of results considering objectives, limitations, multiplicity of analyses, results from similar studies, and other relevant evidence | Line 288-297 |
| Generalisability | 21 | Discuss the generalisability (external validity) of the study results | Line 288-297 |
| Other information | |  | |
| Funding | 22 | Give the source of funding and the role of the funders for the present study and, if applicable, for the original study on which the present article is based | Line 318 |

*Give information separately for cases and controls in case-control studies and, if applicable, for exposed and unexposed groups in cohort and cross-sectional studies.

**Note:** An Explanation and Elaboration article discusses each checklist item and gives methodological background and published examples of transparent reporting. The STROBE checklist is best used in conjunction with this article (freely available on the Web sites of PLoS Medicine at http://www.plosmedicine.org/, Annals of Internal Medicine at http://www.annals.org/, and Epidemiology at http://www.epidem.com/). Information on the STROBE Initiative is available at www.strobe-statement.org.
